# Sudden adult death syndrome in m.3243A>G-related mitochondrial disease: an unrecognized clinical entity in young, asymptomatic adults

**DOI:** 10.1093/eurheartj/ehv306

**Published:** 2015-07-17

**Authors:** Yi Shiau Ng, John P. Grady, Nichola Z. Lax, John P. Bourke, Charlotte L. Alston, Steven A. Hardy, Gavin Falkous, Andrew G. Schaefer, Aleksandar Radunovic, Saidi A. Mohiddin, Matilda Ralph, Ali Alhakim, Robert W. Taylor, Robert McFarland, Douglass M. Turnbull, Gráinne S. Gorman

**Affiliations:** ^1^ Wellcome Trust Centre for Mitochondrial Research, Institute of Neuroscience, Newcastle University, Newcastle upon Tyne, UK; ^2^ Cardiothoracic Centre, Freeman Hospital, Newcastle upon Tyne, UK; ^3^ Department of Neurology, Royal London Hospital, London, UK; ^4^ Department of Cardiology, The London Chest Hospital, London, UK; ^5^ Hemel Hemstead Hospital, Hertfordshire, UK; ^6^ East Surrey Hospital, Redhill, UK

**Keywords:** m.3243A>G, Sudden death, Cardiac, Genetic autopsy

## Abstract

**Aims:**

To provide insight into the mechanism of sudden adult death syndrome (SADS) and to give new clinical guidelines for the cardiac management of patients with the most common mitochondrial DNA mutation, m.3243A>G. These studies were initiated after two young, asymptomatic adults harbouring the m.3243A>G mutation died suddenly and unexpectedly. The m.3243A>G mutation is present in ∼1 in 400 of the population, although the recognized incidence of mitochondrial DNA (mtDNA) disease is ∼1 in 5000.

**Methods and results:**

Pathological studies including histochemistry and molecular genetic analyses performed on various post-mortem samples including cardiac tissues (atrium and ventricles) showed marked respiratory chain deficiency and high levels of the m.3243A>G mutation. Systematic review of cause of death in our m.3243A>G patient cohort showed the person-time incidence rate of sudden adult death is 2.4 per 1000 person-years. A further six cases of sudden death among extended family members have been identified from interrogation of family pedigrees.

**Conclusion:**

Our findings suggest that SADS is an important cause of death in patients with m.3243A>G and likely to be due to widespread respiratory chain deficiency in cardiac muscle. The involvement of asymptomatic relatives highlights the importance of family tracing in patients with m.3243A>G and the need for specific cardiac arrhythmia surveillance in the management of this common genetic disease. In addition, these findings have prompted the derivation of cardiac guidelines specific to patients with m.3243A>G-related mitochondrial disease. Finally, due to the prevalence of this mtDNA point mutation, we recommend inclusion of testing for m.3243A>G mutations in the genetic autopsy of all unexplained cases of SADS.

**Translational perspective**

We report striking respiratory chain deficiency and high levels of the m.3243A>G mitochondrial DNA mutation in cardiac muscle from two young asymptomatic adults found dead-in-bed. Our findings suggest this is an unrecognized clinical entity in individuals carrying the m.3243A>G mutation. We have developed new cardiac guidelines for the management of patients with the m.3243A>G mutation. In addition, because of the frequency of this mutation in the population, it should be screened for in all cases of unexplained SADS.

## Introduction

Mitochondrial disorders are a common cause of inherited disease, exhibiting marked phenotypic and genotypic heterogeneity. The most common form of mitochondria disease is due to the pathogenic mitochondrial DNA (mtDNA) point mutation m.3243A>G in the mt-tRNA leucine gene (*MTTL-1*)*.* The prevalence of this mutation in the population is ∼1 in 400,^1,2^ although the reported incidence of clinically manifesting disease due to the mutation ranges from 3.5 to 16.3 per 100 000 in the adult population.^3,4^

The phenotypes associated with the m.3243A>G mutation include clinical syndromes such as mitochondrial encephalomyopathy, lactic acidosis, and stroke-like episodes (MELAS),^5^ chronic progressive external ophthalmoplegia (CPEO),^6^ and maternally inherited deafness and diabetes (MIDD),^7^ though the majority of patients have clinical features that do not fit any of these classifications.^8^ Cardiac muscle involvement, frequently manifesting as hypertrophic cardiomyopathy (HCM), may occur in up to 40% of patients with m.3243A>G-related mitochondrial disease^9^ and is recognized as an independent predictor of morbidity and early mortality in these patients.^10,11^

The m.3243A>G mutation is invariably heteroplasmic with a mixture of mutated and wild-type mtDNA present in patients. Many patients with high levels of the heteroplasmic m.3243A>G mutation have progressive, disabling disease leading to early death, while individuals with low level m.3243A>G may never develop symptoms. Previous reports of mortality in patients with m.3243A>G mutation predominantly include those symptomatic individuals with chronic and severe disease burden. The most common causes of death include cardiac events;^12^ status epilepticus, stroke-like episodes; aspiration pneumonia or sepsis; paralytic ileus and metabolic acidosis.^13,14^

This study was initiated by the death of two young, asymptomatic m.3243A>G adults who died suddenly and unexpectedly. We believe that these cases represent an important unrecognized clinical entity of m.3243A>G disease. We sought to determine the likely mechanism of the sudden death and the incidence of sudden adult death syndrome (SADS) over the last 5 years in patients known to harbour the m.3243A>G mtDNA point mutation and develop new clincial guidelines to minimize cardiac risk associated with having this mutation.

## Methods

### Index case reports

#### Case 1

A 30-year-old man was assessed clinically following pedigree tracing of m.3243A>G disease. His mother presented with MELAS at the age of 54 and was found to harbour high levels of the m.3243A>G mutation (66% in skeletal muscle, 53% in urine, and 20% in blood). The young man was well, worked full time, and was undertaking regular exercise, including both resistance and endurance training. He was asymptomatic but, on specific questioning, admitted to mild hearing difficulty and occasional mild symptoms similar to irritable bowel syndrome. His routine cardiac investigations including resting 12-lead electrocardiogram (ECG), 24-h ambulatory ECG monitoring, and echocardiography revealed no abnormalities. Magnetic resonance imaging (MRI) brain revealed mild cerebellar atrophy, although neurological examination was normal. Genetic investigations showed 92 and 39% m.3243A>G mutation load in urine and blood, respectively. He had been socializing with friends the night before his death and had consumed 10 units of alcohol. He was found dead-in-bed the following morning.

#### Case 2

A 33-year-old woman was originally identified on pedigree tracing as a member of a family with m.3243A>G disease. Her sister presented in her teens with multiple stroke-like episodes compatible with MELAS and had high levels of m.3243A>G mutation. Her mother and another sister are asymptomatic (m.3243A>G heteroplasmy levels not available). The patient worked full time as a teacher and lived an active life, including going to a gym on a regular basis. She was found to have a normal resting 12-lead ECG and only mild, non-progressive left ventricular hypertrophy (LVH) detected on regular surveillance echocardiography. She reported no cardiorespiratory symptoms. She was taking regular food supplements including Co-enzyme Q10, B-Complex vitamins, and probiotics only. Her m.3243A>G mutation load was 68% in urine and 30% in blood. She was found dead-in-bed at home following a night out with friends.

#### Post-mortem muscle histology and histochemistry

Standard histological and histochemical analyses were performed on fresh frozen 10 µm cryosections from various post-mortem samples including cardiac tissues (atrium and ventricles), skeletal muscle, liver, and kidney. Standard methods included sequential cytochrome *c* oxidase (COX)/succinate dehydrogenase (SDH) histochemistry to assay both Complex IV (COX) and Complex II (SDH) activities.^15^ Sequential COX/SDH histochemistry was performed on frozen 15-µm tissue sections as previously described.^16^ Neuropathological staining and immunohistochemistry was performed on 5 µm sections as previously described.^17^

#### Molecular genetic studies

Total DNA was extracted from several tissues (*Table 1*; see Supplementary material online, *Table S1*) by standard procedures. Pyrosequencing was used to quantify the m.3243G>A heteroplasmy levels.^20^

**Table 1:** A summary of the morphological, histochemical, and molecular genetic findings in cardiac tissue of Cases 1 and 2

	Case 1	Case 2
Body weight (kg)	66	58
Height (m)	1.66	1.63
Autopsy interval (h)	187	96
Toxicology	Negative for illicit drugs Blood ethanol content 63 mg/dL	Negative for illicit drugs and ethanol
Cardiovascular system		
Heart weight (g); Reference: male 360 ± 75; female 308 ± 79	325	365
Gross appearance	Normal	Mild LVH; minimal subendocardial fibrosis of the left ventricular outflow tract
Histopathological findings	Normal	Patchy but prominent cytoplasmic vacuolation and enlargement of cardiac myocytes seen in left ventricle
Histochemistry	40–60% COX deficiency in both ventricles and interventricular septum	15–20% COX deficiency in both ventricles
m.3243A>G Heteroplasmy (%)	Left ventricle: 91 Right ventricle: 95 Interventricular septum: 92 Both atria: 93	Left ventricle: 76 Right ventricle: 78

The reference range of organ weight is based on the findings from these reports.^18,19^

COX, cytochrome c oxidase; RRF, ragged red fibres; LVH, left ventricular hypertrophy; h, hours.

### Cause of death in m.3243A>G patients consented to MRC cohort in Newcastle

Patients identified as harbouring the m.3243A>G mtDNA point mutation and who consented to the Medical Research Council (MRC) Centre Mitochondrial Disease Patient Cohort were identified. We also obtained detailed family histories of those included in the cohort. Cause of death over the last 5 years (April 2009 to October 2014 inclusive) was reviewed and annual incidence-based mortality was calculated. Clinical investigations were performed according to the Declaration of Helsinki. Local study approval was granted (NRES Committee North East- Newcastle & North Tyneside 2) and written informed consent from both patients' families was obtained prior to study inclusion.

### COX (proportional hazards) regression analysis for the development of cardiomyopathy in patients with m.3243A>G mutation

For patients with mitochondrial disease, the dominant cardiac manifestation we have seen is increased LV-wall thickness. This is probably caused by an abnormal, primitive response to energy imbalance in the heart. To define hypertrophy, we have used the standard echo normal range values (defined as diastolic, interventricular septal thickness, and/or diastolic, posterior wall thickness >1.2 cm on 2D transthoracic echocardiography as per British Society of Echocardiography guidelines to distinguish normal from abnormal) and then described as to whether the pattern of hypertrophy seen was septal only or concentric (global). The association of putative factors for the development of cardiomyopathy (gender and heteroplasmy measured in blood, urinary sediment, and skeletal muscle) were investigated by Cox regression^21^ using PROC PHREG in SAS 9.2 (Cary, NC, USA). Statistical significance was determined at *P* < 0.05.

## Results

### Histology and histochemical findings

**Figure 1:**
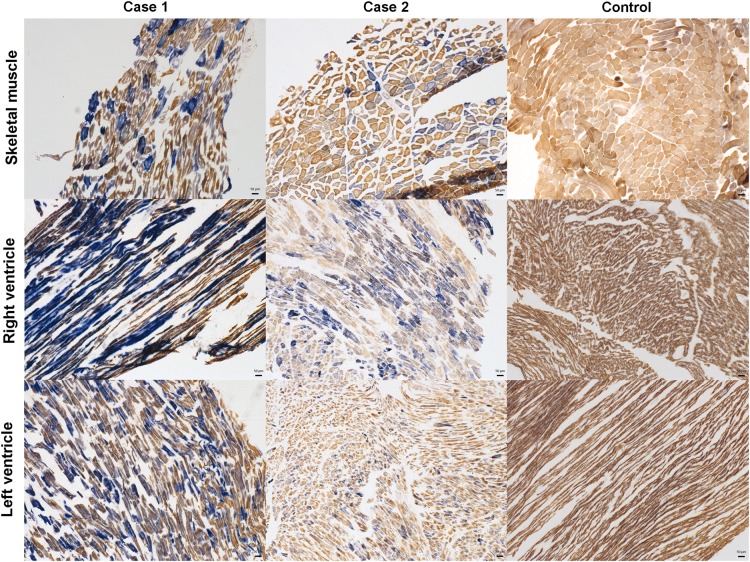
Evidence of mitochondrial dysfunction in skeletal and cardiac muscles by cytochrome *c* oxidase/succinate dehydrogenase histochemistry. To measure cytochrome *c* oxidase/succinate dehydrogenase enzyme activity, sequential cytochrome *c* oxidase/succinate dehydrogenase histochemistry was performed on post-mortem skeletal (quadriceps) and cardiac (right and left ventricle) muscle sections from patients and a control. Cells that are identified by a brown reaction product have functional cytochrome *c* oxidase and succinate dehydrogenase activity; those that are blue have lost cytochrome *c* oxidase activity but retain succinate dehydrogenase activity. This assay reveals a mosaic pattern of variable cytochrome *c* oxidase-deficiency (blue cells) within all patients' tissues (Case 1 and 2), while control tissues (Control) reveal functional cytochrome *c* oxidase and succinate dehydrogenase activities (brown cells). Scale bar = 50 μm.

Drug toxicology screen was negative for both Case 1 and 2, while low level of alcohol (63 mg/dL) was detected in Case 1. There was no gross structural cardiac abnormality found in Case 1, and there was only mild left ventricular hypertrophy identified in Case 2. The cause of death could not be determined for either case at initial autopsy. The autopsy findings, tissue morphology, histochemistry, and heteroplasmy level of both cases are summarized in *Table 1* and see Supplementary material online, *Table S1*. Histochemical analyses of cardiac tissue showed high levels of COX deficiency in both cases (*Figure 1*). There was additional patchy but prominent cytoplasmic vacuolation and enlargement of cardiomyocytes in Case 2’s left ventricle. There was limited evidence of CNS involvement in either case with neuronal population density intact with an absence of astrogliosis and ischaemic lesions, although some evidence of respiratory chain deficiency particularly in blood vessels.

### Levels of heteroplasmy of m.3243A>G mutation in tissues

The level of m.3243A>G was high in all examined tissues from Case 1 with over 90% in several cardiac tissues. There was also high level of m.3243A>G in brain, liver, and adrenal glands. In Case 2, levels of m.3243A>G were also high in multiple tissues including heart muscle and brain (see Supplementary material online,Supplementary Data).

### Cause of death in m.3243A>G patients consented to MRC cohort Newcastle

We included 209 patients with the m.3243A>G mutation; there were 14 deaths documented over the preceeding 6 years (April 2009 to October 2014 inclusive) (*Table 2*). We estimate that the incidence rate of all-cause mortality for m.3243A>G patients is 17 per 1000 person-years (95% CI 9.1–28) and the incidence rate of SADS is 2.4 per 1000 person-years (95% CI 0.29–8.6)(see Supplementary material online,Supplementary Data). Interrogation of family pedigrees of these 209 patients (90 pedigrees) identified a further 6 adults (18–44 years) who were clinically asymptomatic or mildly affected but presumed obligate carriers, as having died suddenly and unexpectedly (prior to the establishment of the MRC cohort), suggesting that SADS is not uncommon but perhaps, until now, an unrecognized clinical entity in young, asymptomatic adults with m.3243A>G disease.

**Table 2:** Cause of death in patients harbouring the m.3243A>G mutation in Newcastle from April 2009 to October 2014

No	Sex	Deceased age	Heteroplasmy			NMDAS score	Cause of death
			Mu (%)	U (%)	B (%)		
1	M	48		55	13	19	Coronary artery disease^a^
2	F	65	62	60	17	62	HCM
3	M	12		82	46	N/P	HCM and renal failure
4	M	58	70			N/P	Not available^a^
5	M	60		33	7	50	Aspiration pneumonia secondary to bowel pseudo-obstruction
6	F	61		45	7	0	Metastatic breast cancer^a^
7	M	20		93	66	19	Cardiorespiratory failure (HCM)
8	M	23		93	61	67	Multi-organ failure
9	M	33		90	3	65	End-stage MELAS
10	F	53		91		49	Severe encephalopathy secondary to urinary tract infection
11	F	66	39	55	6	60	Aspiration pneumonia secondary to bowel pseudo-obstruction
12	M	52		75	29	42	HCM and pneumonia
13	F	33	90	68	30	4	SADS^b^
14	M	30	85	53	20	4	SADS^b^

The mean deceased age is 43.5 years with 95% CI (33.2–53.8).

M, male; F, female; Mu, muscle; U, urine; B, blood; NMDAS, Newcastle Mitochondrial Disease Adult Scale; HCM, hypertrophic cardiomyopathy; MELAS, mitochondrial encephalomyopathy, lactic acidosis and stroke-like episode; SADS, sudden adult death syndrome.

^a^Unrelated to mitochondrial disease.

^b^Two index cases of this report.

### Identifying associated factors for development of cardiomyopathy in patients with m.3243A>G mutation

**Figure 2:**
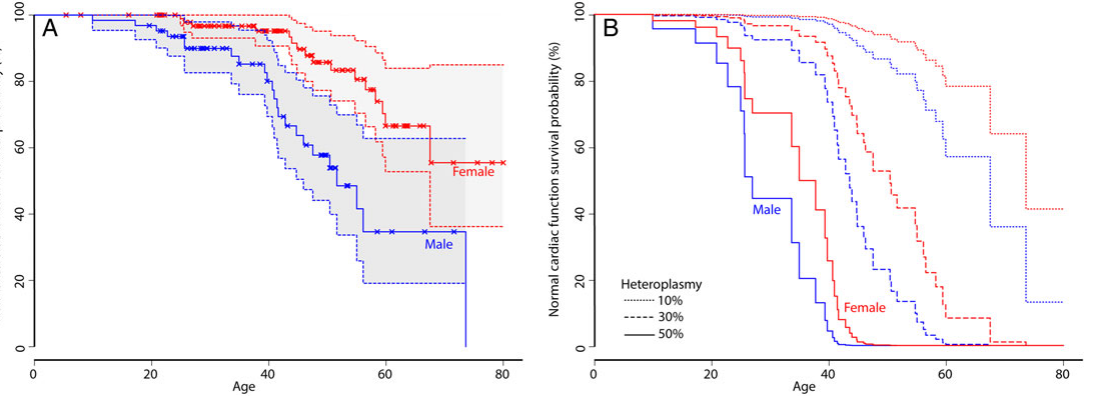
Normal cardiac function survival probability according to gender and blood heteroplasmy. (*A*) Normal cardiac function survival probability (survival function) for males (blue) and females (red). Censored data (i.e. patients who have not developed cardiomyopathy at their most recent clinical assessment) are shown as crosses, normal cardiac functions are shown as solid lines with 95% confidence intervals. The age for developing cardiomyopathy in males is significantly younger than females (*P* = 0.0143). (*B*) Normal cardiac function survival probability (survival function) for males (blue) and females (red) with varying levels of blood heteroplasmy (10% dotted lines, 30% dashed lines, 50% solid lines).

Median survival time to the development of cardiomyopathy for all patients is 67.6 years. Using Cox regression, we identified that the most explanatory factors for the development of cardiomyopathy are blood heteroplasmy (*P* < 0.0001) and gender (*P* = 0.0143) (*n* = 164), with men significantly at greater risk for the development of cardiomyopathy (for details, see Supplementary material online,Supplementary Data). Survival functions using blood heteroplasmy and gender are shown in *Figure 2* and demonstrate a male predilection to earlier onset of cardiomyopathy (*P* = 0.0143). These data have facilitated the development of a simple, on-line tool for plotting the prediction of the development of cardiomyopathy for a given heteroplasmy and gender (http://research.ncl.ac.uk/mitoresearch/cardio/).

## Discussion

We believe these two patients presenting with SADS highlight an important and unrecognized phenotype in those who carry the m.3243A>G mutation. There are several striking similarities between the cases: (i) they were only identified as carrying the m.3243A>G mutation via family screening and were asymptomatic; (ii) they had high mutation load in both urine and blood; (iii) there was extensive evidence of respiratory chain deficiency in multiple organs and especially in heart; (iv) at least one of their immediate family members clinically manifested with the severe MELAS phenotype; (v) both were last seen in good health within 12 h of their sudden death.

Our centre's experience of all-cause mortality in patients with m.3243A>G disease is similar to that reported by other groups. However, sudden death in patients with m. 3243A>G mutation is rarely reported in the literature, and it appears to occur among those with severe disease, and identifiable risk factors including cardiomyopathy, diabetes, and epilepsy, that could plausibly have contributed to their deaths.^11,14^ These unexpected deaths in our patients suggests that SADS can occur without identifiable risk factors^11^; as in Case 1, and across the entire spectrum of m.3243A>G disease, from asymptomatic to severely affected individuals.

Given the circumstances and lack of any prodrome, the likely cause of sudden death in our patients is a cardiac arrhythmia. Cardiac abnormalities are the main cause of sudden death in young adults. HCM is common in patients harbouring the m.3243A>G mutation.^22^ In addition, cardiac arrhythmias have also been described frequently in patients with m.3243A>G including Wolff–Parkinson–White syndrome, supraventricular tachycardia, atrial fibrillation, and depolarization abnormalities.^12,23,24^ Cardiac fibre disarray that has been shown to underlie ventricular tachyarrhythmias in some forms of HCM^25,26^ was not evident in our two cases. Case 1 had a normal resting ECG, echocardiogram, and 24-h ECG monitoring. His autopsy did not reveal any gross structural cardiac abnormality. Case 2 had documented mild LVH; however, the extent of left ventricular wall thickness index would not have suggested an increased risk of sudden cardiac death.^27^ Perhaps most surprising was the lack of cardiac manifestations considering the severity of the biochemical defect found in cardiomyocytes of both patients. Though neither patient had consumed large amounts of alcohol, or appeared unwell to their friends, extreme sensitivity to alcohol has previously been reported in patients with respiratory chain disease.^28^ A potential theoretical risk is that lactic acidosis caused by the combined effect of the underlying cardiac respiratory chain defect, with the inhibitory effects of alcohol on gluconeogenesis,^29^ may have triggered a fatal cardiac arrhythmia and would advise caution in the use of alcohol in this group of patients. However, given that many of these patients develop lactic acidosis without suffering from cardiac arrhythmias, we believe that further work on this aspect is required.

Death related to seizures is also a possibility. Seizure-induced central hypoventilation, cardiac arrhythmia, or abnormal autonomic response have been proposed as possible mechanisms in sudden unexplained death in epilepsy (SUDEP).^30^ However, neither patient had a history of epilepsy, and brain structure was intact at autopsy, though it did show evidence of respiratory-deficient neurons and blood vessels. Whether this makes the patients more vulnerable to seizures and sudden death is not known.

It is also possible that the deaths are unrelated to the m.3243A>G mutation, though no other cause of death was identified at autopsy. Sudden death in young adults is very rare and the incidence ranges from 1 to 3.73 per 100 000.^31–34^ The majority of these deaths have a cardiac aetiology with up to 70% having structurally abnormal hearts at autopsy.^33,35^

**Figure 3:**
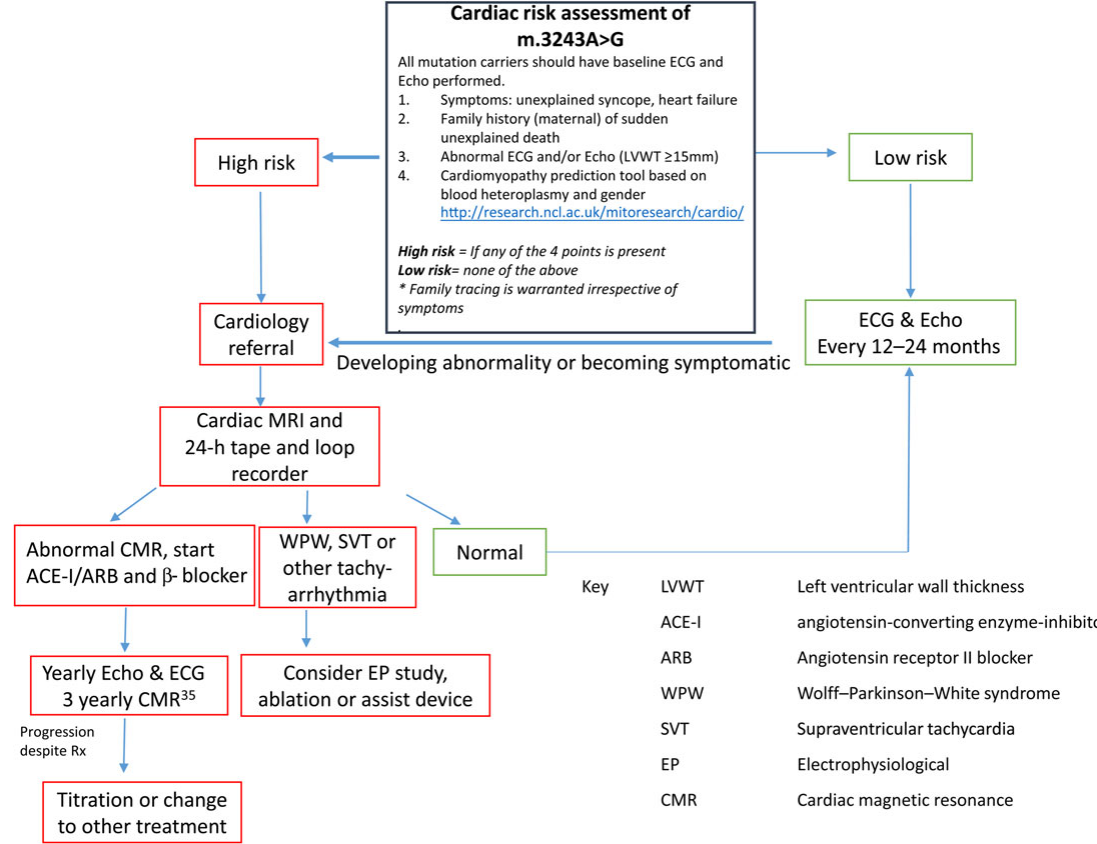
Summary flow diagram of cardiac risk assessment and management of patients harbouring the m.3243A>G mutation.

These cases highlight again the challenges of developing cardiac care pathways for patients with m.3243A>G mutation. The recently devised tool (HCM Risk-SCD)^36^ to predict sudden death in HCM, unfortunately would not have identified either of our patients at risk of sudden death, emphasizing the complexity of these metabolic disorders. Based on these guidelines, the data presented, and our centre's clinical experience, we propose the following Expert Opinion Guidelines (summarized in *Figure 3*):

1. Patients with ≥15 mm left ventricular wall thickness index on echocardiogram^27^ and/or evidence of a pre-excitation syndrome on ECG and/or family history of sudden adult death syndrome should be made aware of the risk of sudden death and the importance of regular health surveillance.^37^
2. All individuals who harbour the m.3243A>G mutation should have a cardiovascular risk assessment. We advise implementation of the prediction tool to stratify individual risk of cardiomyopathy development in patients with m.3243A>G-related mitochondrial disease (http://research.ncl.ac.uk/mitoresearch/cardio/). High-risk individuals are defined as those having normal cardiac function survival probability of 95% or lower for a given blood heteroplasmy and gender and should be offered cardiac magnetic resonance (CMR) to better define the presence of and/or degree of LVH more accurately.^37,38^ Early introduction of a combination of angiotensin-converting enzyme inhibitor (ACE-I) and β-blocker for those with confirmed cardiomyopathy is advocated routinely by our centre, based on best practice cardiac guidelines.^39^
3. Cardiac electrophysiology testing is advisable for those with overt or suspected pre-excitation syndromes (e.g. WPW syndrome) or AV-conduction abnormalities (e.g. prolonged PR interval or prolonged QRS duration), particularly in those with palpitations or unexplained episode/s of collapse. We now also recommend implantation of a ECG loop recorder to provide longer term surveillance of arrhythmias in patients at high risk determined on blood heteroplasmy and age (see above) and/or ≥15 mm left ventricular wall thickness index on echocardiogram. Accessory pathways capable of sustaining arrhythmias and those with short antegrade refractory periods should undergo catheter ablation. Those confirmed to have episodes of non-sustained ventricular tachycardia on loop-recorder surveillance should be considered for a cardioverter-defibrillator.^37,39^ Those with confirmed pathological bradycardia due to either sinus or AV-nodal conduction delay can be considered for pacemaker fitting.
4. Patients harbouring the m.3243>G mutation and with documented epilepsy should be advised of the inherent risk of SUDEP.
5. There should be extensive family tracing and genetic testing offered to the relatives of patients with m.3243A>G since even asymptomatic family members are at risk of sudden death.

We acknowledge that there are inherent limitations with aspects of these guidelines, namely: In conclusion, we report findings from two asymptomatic, young adults with high levels of the m.3243A>G mutation who died suddenly and unexpectedly. The autopsy findings strongly point to an arrhythmia mechanism of death. Combined with an annual incidence of 2.4 per 1000 person-years, these findings suggest that SADS is not uncommon. Most importantly, it is an unrecognized clinical entity in young, asymptomatic adults with m.3243A>G disease. This suggests the need for increased vigilance in the cardiac monitoring of even apparently asymptomatic individuals with the m.3243A>G mutation. More detailed investigation and increased deployment of implantable ECG loop recorders are justified by the need to develop better predictors of arrhythmia risk.

1. Several aspects of our proposed guidelines (points 1 and 2) as markers of SAD risk are made based upon extrapolation from HCM. To date, we do not know whether identical limits apply in m.3243A>G-related mitochondrial disease; aspects that require further elucidation.
2. Although these are expert opinion guidelines from a single centre, we are a nationally commissioned multispecialty and multidisciplinary service, charged with the care of patients with mitochondrial disease from throughout the UK, exemplified by our patient cohort of over 700 patients, which is the part of the largest cohort worldwide. Combined with over 25 years' experience in this field, this places us in a unique position to devise such guidelines.
3. Lastly, the incidence of cardiomyopathy on echocardiogram maybe an under estimation of the true incidence of cardiac involvement as we have previously shown.^38^

In light of our findings, we recommend comprehensive family tracing to screen for mitochondrial disease and where possible, histochemical and/or molecular testing in all index cases of SADS. Relatively simple additional investigations on autopsy samples would screen for the m.3243A>G mutation and for abnormal respiratory chain function in cardiac and skeletal muscle using COX/SDH histochemistry.

## Supplementary material

Supplementary material is available at *European Heart Journal* online.

## Funding

This work was supported by The Wellcome Trust (074454/Z/04/Z, http://www.newcastle-mitochondria.com/), Newcastle University Centre for Ageing and Vitality (supported by the Biotechnology and Biological Sciences Research Council and Medical Research Council [M501700]), UK NIHR Biomedical Research Centre for Ageing and Age-related disease award to the Newcastle upon Tyne Hospitals NHS Foundation Trust, Lily Foundation and the UK NHS Specialist Commissioners which funds the ‘Rare Mitochondrial Disorders of Adults and Children’ Diagnostic Service in Newcastle upon Tyne. This work also received infrastructure support from the Newcastle NIHR Biomedical Research Centre, Newcastle and North Tyneside Comprehensive Local Research Network. Funding to pay the Open Access publication charges for this article was provided by the Wellcome Trust Centre 096919/Z/11/Z.

**Conflict of interest:** none declared.

## Supplementary Material

Supplementary Data
