# Supplementary material for: Sudden adult death syndrome in m.3243A>G-related mitochondrial disease: an unrecognized clinical entity in young, asymptomatic adults
Source: Eur Heart J. 2015 Jul 17;37(32):2552–9. doi: 10.1093/eurheartj/ehv306 (PMC5008417; doi:10.1093/eurheartj/ehv306)
Supplement: Supplementary Data [file ehv306_supplementary_data.zip › ehv306supp_table1.docx]

**Supplemental data** – **Sudden Adult Death Syndrome in m.3243A>G-related mitochondrial disease: an unrecognised clinical entity in young, asymptomatic adults**

**Supplemental Table 1.** A summary of the autopsy findings including details related to non-cardiac tissue morphology, histochemistry and heteroplasmy. COX= cytochrome c oxidase; RRF= ragged red fibres; GI= gastrointestinal; N/A= not available. The reference range of organ weight is based on the findings from these reports[^39^](#_ENREF_39)^,^ [^40^](#_ENREF_40).

|  | Case 1 | Case 2 |
| --- | --- | --- |
| **Respiratory system** | | |
| **Lung weights (g)**  *Reference: male right lung 625 ± 307; male left lung 551 ± 178; female right lung 545 ± 183; female left lung 472 ± 181* | Left lung: 608  Right lung: 625 | Left lung: 473  Right lung: 665 |
| **Appearance** | Both lungs were congested | Both lungs showed pulmonary oedema with some mucus plugging and mild hyperinflation |
| **Gastro-intestinal system** | | |
| **Appearance**  *Liver weight reference: male 1637 ± 369g; female 1496 ± 331g* | Stomach was full of undigested food (600ml). Normal upper and lower GI tracts; liver weighed 1412g and pancreas was normal | Normal upper and lower GI tracts; liver weighed 1732g and there was no focal lesion in pancreas |
| **Skeletal muscle** | | |
| **Histochemistry** | 20% COX deficiency | 25% COX deficiency |
| **Heteroplasmy (%)** | 85 | 90 |
| **Central nervous system** | | |
| **Brain Weight (g)**  *Reference: Male 1440g; Female 1290g* | 1168 | 1438 |
| **Appearance** | No evidence of haemorrhage or infarction | No haemorrhage, tumour or focal lesion in cerebrum, cerebellum and brainstem |
| **Histochemistry** | Few COX-deficient neurons and blood vessels in temporal cortex | Multiple COX-deficient neurons and blood vessels with patchy COX-deficiency |
| **Heteroplasmy (%)**  Frontal cortex  Temporal cortex  Cerebellum | N/A  90  N/A | 85  85  79 |
| **Other organs** | | |
| **Heteroplasmy (%)**  Liver  Kidney  Adrenal gland | 86  N/A  90 | 82  85  N/A |
