# Supplementary material for: Sudden adult death syndrome in m.3243A>G-related mitochondrial disease: an unrecognized clinical entity in young, asymptomatic adults
Source: Eur Heart J. 2015 Jul 17;37(32):2552–9. doi: 10.1093/eurheartj/ehv306 (PMC5008417; doi:10.1093/eurheartj/ehv306)
Supplement: Supplementary Data [file ehv306_supplementary_data.zip › ehv306supp_table3.docx]

**Supplemental data – Ng et al. Sudden Adult Death Syndrome in m.3243A>G-related mitochondrial disease: an unrecognised clinical entity in young, asymptomatic adults**

**Supplemental table 3**. Cox proportional hazards survival regression analysis. For heteroplasmy the hazard ratio relates to a 10% change in heteroplasmy, for sex it relates to the increased hazard associated with males.

|  | | Heteroplasmy | | Gender | |
| --- | --- | --- | --- | --- | --- |
| Heteroplasmy  source | **N** | **P value** | **Hazard Ratio** | **P value** | **Hazard ratio** |
| Blood | 164 | < 0.0001 | 2.569  (1.901 – 3.473) | 0.0143 | 2.304  (1.182 – 4.493) |
| Urine | 172 | 0.0036 | 1.286  (1.086 – 1.523) | 0.0465 | 2.072  (1.011 – 4.245) |
| Muscle | 54 | 0.4241 | 1.130  (0.838 – 1.523) | 0.7222 | 0.821  (0.276 – 2.442) |
