# Supplementary material for: Sudden adult death syndrome in m.3243A>G-related mitochondrial disease: an unrecognized clinical entity in young, asymptomatic adults
Source: Eur Heart J. 2015 Jul 17;37(32):2552–9. doi: 10.1093/eurheartj/ehv306 (PMC5008417; doi:10.1093/eurheartj/ehv306)
Supplement: Supplementary Data [file ehv306_supplementary_data.zip › ehv306supp_table2.docx]

**Supplemental data – Ng et al. Sudden Adult Death Syndrome in m.3243A>G-related mitochondrial disease: an unrecognised clinical entity in young, asymptomatic adults**

**Supplemental table 2**. Number of patients with m.3243A>G followed up in Newcastle between April 2009 to Oct 2014

| Year | No of patients recruited/ year | No of patients deceased/ year | Cumulative number of alive patients |
| --- | --- | --- | --- |
| 2009 | 61 | 1 | 60 |
| 2010 | 39 | 0 | 99 |
| 2011 | 33 | 2 | 130 |
| 2012 | 41 | 2 | 169 |
| 2013 | 23 | 6 | 186 |
| 2014 | 12 | 3 | 196 |
| Total | 209 | 14 | 840 |

Total number of deaths over 6-year period = 14

Person-year of observation over 6-year period = 60 + 99 + 130 + 169 + 186 + 196

= 840

**Incidence of all deaths** = 14/840

= 0.017 death per person-year

= 17 per 1000 person-years

**Incidence of SADS** = 2/840

= 0.0024 death per person-year

= 2.4 per 1000 person-years
